# Providers adherence to essential contents of antenatal care services increases birth weight in Bahir Dar City Administration, north West Ethiopia: a prospective follow up study

**DOI:** 10.1186/s12978-018-0610-8

**Published:** 2018-09-29

**Authors:** Tadese Ejigu Tafere, Mesganaw Fanthahun Afework, Alemayehu Woreku Yalew

**Affiliations:** 1 School of Public Health (SPH), College of Health Sciences, Addis Ababa University, Addis Ababa, Ethiopia; 2 School of Public Health, College of Medicine and Health Sciences, Bahir Dar University, Bahir Dar, Ethiopia

**Keywords:** Quality of antenatal care, Birth weight, Pregnancy outcome, Neonatal mortality

## Abstract

**Background:**

Low birth weight (LBW) is one of the most important factors affecting child morbidity and mortality worldwide. Antenatal care (ANC) is an opportunity for reaching pregnant women with a number of interventions that may be vital to their health and well-being of their infants. However, data on the link between ANC quality and LBW remain limited especially in developing countries. Therefore, this study was aimed at investigating the effect of ANC service quality on birth weight among pregnant women attending ANC at public health facilities of Bahir Dar City Administration, Bahir Dar, Ethiopia using provision of essential services by providers as proxy for quality of care.

**Methods:**

Nine hundred seventy pregnant women with gestational age ≤ 16 weeks who came for their first ANC visit and selected by systematic sampling were enrolled and followed until delivery. Longitudinal data was collected during consultation with ANC providers using structured observation checklist. Women who gave birth at home and those who deliver a premature or still birth baby were excluded as data on birth weight could not be obtained for home deliveries and as the birth weight of the baby might be affected due to prematurity and still birth. Completed data were obtained from 718 women (since the rest women gave birth at home, we could not obtain birth weight data and we exclude them from analysis). The overall ANC service was considered as acceptable quality if women received ≥75th percentile of the essential ANC services. Generalized Estimating Equation was carried out to identify predictors of birth weight by controlling the cluster effect among women who received ANC services in the same facility.

**Results:**

The prevalence of low birth weight (< 2500 g) was 7.8% (95%CI = 6.0%, 9.7%) with 1.4% versus 10.5% among those who received acceptable and not acceptable quality ANC services respectively, *P*-value< 0.001. Maternal nutritional advice, iron-folic acid supplementation, tetanus toxoid vaccination, maternal educational status, parity and age were determinants for birth weight.

**Conclusion and recommendation:**

The study showed that access to quality ANC services led to good birth weight outcome. Strengthening adherence of providers to essential components of antenatal care through regular monitoring and need based capacity building is very important for reducing the risk of low birth weight.

## Plain English summary

A child’s birth weight or size at birth is an important indicator of the child’s vulnerability to the risk of childhood illnesses and the child’s chances of survival. Low birth weight (LBW) contributes to 60% to 80% of all neonatal deaths. Though this is the fact, based on the 2016 Ethiopian demographic and Health survey report, the prevalence of LBW in Ethiopia is 13.4%. It is also worth noting the prevalence might be higher as only 14% of children in Ethiopia are weighed at birth. However, data on the link between ANC quality and LBW remain limited especially in low income countries like Ethiopia.

Pregnant women with gestational age ≤ 16 weeks who came for their first ANC visit and selected by systematic random sampling were enrolled and followed until delivery. Longitudinal data were collected during consultation with ANC providers to assess the quality of ANC services during their four focused ANC visits using structured observation checklist. The overall ANC service was considered as acceptable quality if women received ≥75th percentile of the essential ANC services.

Among 970 enrolled pregnant women, completed data for this study was obtained from 718 women (as the rest women give birth at home they are excluded from the analysis due to lack of data on birth weight).

The prevalence of low birth weight (< 2500 g) for the current study was 7.8% (95%CI = 6.0%, 9.7%) with 1.4% versus 10.5% among those who received acceptable and not acceptable quality ANC services respectively. This indicates quality ANC service has a better birth weight outcome, that implied a woman’s contact with her ANC provider should be more than a simple visit.

## Background

The World Health Organization (WHO) has defined low birth weight (LBW) as the weight of live born infants of < 2500 g regardless of gestational age or any other factor that might have an influence on it. A child’s birth weight or size at birth is an important indicator of the child’s vulnerability to the risk of childhood illnesses and the child’s chances of survival. The global prevalence of LBW is in the range between 15 and 20%, which accounts to about 20 million infants born; approximately one third of neonatal deaths are attributable to LBW [1].

The prevalence of LBW in Ethiopia and Amhara region in particular was 11 and 11.2% respectively. It is also worth noting these rates might be higher as only 5% of children in Ethiopia are weighed at birth [2]. The goal set by WHO is to achieve a 30% reduction of the number of infants born with a weight < 2500 g by the year 2025; 3.9% relative reduction per year between 2012 and 2025 [3].

Antenatal care (ANC) is an opportunity for reaching pregnant women with a number of interventions that may be vital to their health and well-being of their infants [4]. Its putative benefits to babies include increased intra uterine growth, reduced risk of infection and increased survival [5].

The assessment of prenatal care uptake and quality are essential steps toward improving accessibility and birth outcomes. Some elements of the ANC package (Iron –folic acid supplementation, nutritional advice, tetanus toxoid vaccination, screening for pre-eclampsia, screening and treatment of asymptomatic bacteriuria and syphilis) have been shown to be cost-effective in a Sub-Saharan African context [6, 7].

Even though the prevalence of LBW is higher in low and middle income countries, most of the research on the link between antenatal care and birth weight has been conducted in high income nations [1] and many of these were cross sectional studies that focus on assessing the association between frequency (uptake) of antenatal visits rather than adherence of providers to essential services of antenatal care and birth weight. In addition, low income countries usually follow guidelines based on research findings conducted in high income nations. However, the results from research conducted in these countries are not necessarily applicable to low income nations. Therefore, the aim of this study was to investigate the association between adherences of providers to essential services of antenatal care and birth weight which might have an input for the design of guidelines and policies to improve child survival in low income countries like Ethiopia.

## Methods

### Study design, setting and population

A facility based prospective follow up study was conducted from October 2015 to August 2016 in Bahir Dar City Administration, Amhara National Regional State, which is located in the North West part of Ethiopia. According to the Amhara National Regional State Bureau of Finance and Economic Development report, the projected population by 2015/16 was 297,775 (80.5% urban Vs 19.5% rural), of these 156,515 (52.6%) were females and there were 10,035 eligible pregnant women in the same year [8].

The study was conducted on seven public health facilities, one hospital and six health centers. All selected first visit pregnant women with gestational age ≤ 16weekss and attending ANC service during data collection period that were voluntary to participate verbal informed consent were included in the study. Women who gave birth at home were excluded as data on birth weight could not be obtained. In addition, those who gave a premature or still-birth baby; those who had any medical complication while pregnant (diabetes mellitus, heart disease, eclamptic and multiple pregnancy or give birth by caesarian section) were excluded as the birth weight of the baby might be affected due to the underlying conditions.

### Sample size and sampling technique

The current study is part of a large follow up study with multiple objectives. The detail of the sample size calculation assumptions to address all objectives is described in the other part of the study published on Plos ONE [9]. For the current study a sample size was calculated with the predicted incidence of LBW babies as 6% among mothers completing four antenatal visits (compared to 25% among mothers with less than four visits) [10], and with 80% power, 95% confidence level and 15% non-response rate. The final calculated sample size was 304. However, to increase the power of the study, all 718 women who deliver a singleton baby at the study health facilities were included in the analysis.

### Data collection procedure

A structured observation check list to measure ANC quality was developed based on focused antenatal care protocol and used to observe the routine ANC practice for each visit. The weight of naked neonates was measured in kilograms to the nearest 100 g by using standard beam balance in the delivery room immediately after birth (preferably within 1 hour) by trained diploma midwives. Seven female diploma Midwives and two Bachelor of Science female Midwives were recruited as data collectors and supervisors respectively. Both data collectors and supervisors were not working in health facilities under the study. Training was given on the data collection instrument and how to approach and observe the ANC service provision. Pretest of the instrument, close supervision and daily checkup of the filled questionnaire for completeness were also done to maintain the data quality.

#### Dependent variable

Birth weight in kilograms; which was measured within 1 hour after birth.

#### Independent variable

Quality of ANC service was the main exposure variable.

Quality of ANC service was measured based on the interventions that the mother received during antenatal care: laboratory tests done like blood and urine tests, physical examinations like weight, height, blood pressure, providing TT vaccination, iron - folic acid supplementation, deworming, nutritional advice, information on immunization and breast feeding.

If the essential ANC service was given it was coded as 1 otherwise 0 and a composite index for the overall ANC service quality was calculated. Percentile was computed to categorize the quality of the services a woman received. If she scored ≥75th percentile, of essential ANC services, ANC service was considered acceptable quality otherwise not [11].

#### Data analysis

Data were coded and entered into EPI data version3.1 and exported to SPSS version 20 for analysis. Descriptive statistics were used to describe the data. Generalized Estimating Equation linear regression analysis with robust estimator and exchangeable working correlation matrix was carried out to control the cluster effect of women who received ANC services within the same facility and to identify the predictor variables for birth weight of neonates. A *p*-value < 0.2 was considered to retain variables for the multivariable Generalized Estimating Equation linear regression analysis and *P*-value < 0.05 was considered to identify statistically significant predictors for birth weight.

## Results

### Characteristics of ANC attendants

Among 718 women who gave birth at health facilities, those with age ≤ 19 years (teen age pregnancy) and those with age ≥ 35 years (elderly pregnancy) were 4.9 and 4.0% respectively. The mean (±standard deviation) age at birth was 25.32 ± 4.21 years. Above 90 %, (94.2%) of women were urban residents and more than half of them (52.9%) were house wives. Above 90 %, (90.9%) of study participants were Orthodox Christian and almost all women (98.8%) were currently married. With regard to their educational status, 11.4% of women had no formal education while 63.3% of women had joined secondary school and above (Table 1).

**Table 1:** Socio-demographic characteristics and birth weight among study participants in public health facilities of Bahir Dar City administration (*N* = 718), October 2015 to August 2016

	Birth weight			Total frequency
Variables	low	normal	over weight	
Age				
≤ 19	0 (0.0%)	34 (97.1%)	1 (2.9%)	35 (100.0%)
20–24	20 (7.0%)	260 (91.5%)	4 (1.4%)	284 (100.0%)
25–29	16 (5.9%)	245 (9.1%)	8 (3.0%)	269 (100.0%)
30–34	7 (7.0%)	93 (92.1%)	1 (0.9%)	101 (100.0%)
≥ 35	13 (44.8%)	16 (55.2%)	0 (0.0%)	29 (100.0%)
Residence				
Urban	43 (6.4%)	619 (91.6%)	14 (2.1%)	676 (100.0%)
Rural	13 (31.0%)	29 (69.0%)	0 (0.0%)	42 (100.0%)
Educational status				
No formal education	23 (20.9%)	86 (78.2%)	1 (0.9%)	110 (100.0%)
Primary	15 (9.8%)	138 (90.2%)	0 (0.0%)	153 (100.0%)
Secondary	18 (4.0%)	424 (93.1%)	13 (2.9%)	455 (100.0%)
Occupation				
Farmer	13 (32.5%)	27 (67.5%)	0 (0.0%)	40 (100.0%)
House wife	31 (8.2%)	346 (91.0%)	3 (0.8%)	380 (100.0%)
Employee^a^	12 (4.0%)	275 (92.3%)	11 (3.7%)	298 (100.0%)
Religion				
Orthodox	51 (7.8%)	589 (90.2%)	13 (2.0%)	653 (100.0%)
Muslim	5 (7.9%)	57 (90.5%)	1 (1.6%)	63 (100.0%)
Protestant	0 (0.0%)	2 (100.0%)	0 (0.0%)	2 (100.0%)
Marital status				
Married	54 (7.6%)	641 (90.4%)	14 (2.0%)	709 (100.0%)
Divorced	1 (12.5%)	7 (87.5%)	0 (0.0%)	8 (100.0%)
Widowed	1 (100.0%)	0 (0.0%)	0 (0.0%)	1 (100.0%)
Ethnicity				
Amhara	55 (8.1%)	619 (90.2%)	12 (1.7%)	686 (100.0%)
Tigre	1 (4.5%)	19 (86.4%)	2 (9.1%)	22 (100.0%)
Awi	0 (0.0%)	7 (100.0%)	0 (0.0%)	7 (100.0%)
Oromo	0 (0.0%)	3 (100.0%)	0 (0.0%)	3 (100.0%)
Grand total	56 (7.8%)	648 (90.3%)	14 (1.9%)	718 (100.0%)

^a^governmental employees, nongovernmental employees, daily laborers and waiters

Greater than two out of five of the pregnant women, 326 (45.4%) were primi-para; while more than one out of two 378 (52.6%) of women were multi-para and 14 (1.9%) of them were grand multi para.

### Adherence to essential contents of antenatal care and birth weight

Above 95 %, 701(97.6%) of pregnant women received two doses of tetanus toxoid vaccine but the rest 17 (2.4%) missed one dose. About two third (57.1%) of the pregnant women were counselled about BPCRP at least once in their four ANC visits. The information provision on maternal nutrition, breast feeding and immunization was also heterogeneous (Table 2).

**Table 2:** Information provided to pregnant women during ANC visits at public health facilities of Bahir Dar City Administration, Bahir Dar, Ethiopia; October 2015 to August 2016 (*N* = 718)

ANC services given	During 1st visit, n (%)	During 2nd visit, n (%)	During 3rd visit, n (%)	During 4th visit, n (%)
Advised on maternal nutrition	496 (69.1)	608 (84.7)	506 (70.5)	296 (41.2)
Advised on breast feeding	–	–	14 (1.9)	205 (28.6)
Advised immunization	–	–	11 (1.5)	196 (27.3)
Checked conjunctiva for pallor	575 (80.1)	238 (33.1)	507 (70.6)	272 (37.9)

With regard to iron supplementation, 304 (42.3%), 327 (45.5%), 77 (10.7%) and 10 (1.4%) of pregnant women were supplemented with 120, 90, 60 and 30 tablets of iron- folic acid respectively. In addition, 509 (70.9%) and 564 (78.6%) of pregnant women received anti-heliments for deworming and were advised to use insecticide treated bed net for prevention of malaria respectively (Table 3).

**Table 3:** Birth weight status of neonates among pregnant women who received different ANC services at public health facilities of Bahir Dar City Administration, Bahir Dar, Ethiopia; October 2015 to August 2016 (*N* = 718)

Variables		Low birth weight, n (%)	Normal birth weight, n (%)	Overweight, n (%)	Total, n (%)
Quality of ANC	Received acceptable quality	3 (1.4%)	203 (94.4%)	9 (4.2%)	215 (100.0%)
	Received not acceptable quality	53 (10.5%)	445 (88.5%)	5 (1.0%)	503 (100.0%)
Maternal nutritional advice	Not advised at all	4 (13.3%)	25 (83.3%)	1 (3.3%)	30 (100.0%)
	Advised in one visit	7 (53.8%)	6 (46.1%)	0 (0.0%)	13 (100.0%)
	Advised in two visits	30 (11.7%)	225 (87.5%)	2 (0.8%)	257 (100.0%)
	Advised in three visits	12 (4.1%)	275 (93.8%)	6 (2.0%)	293 (100.0%)
	Advised in four visits	3 (2.4%)	117 (93.6%)	5 (4.0%)	125 (100.0%)
Iron - folic acid supplementation	Given 30 tablets	9 (90.0%)	1 (10.0%)	0 (0.0%)	10 (100.0%)
	Given 60 tablets	18 (23.4%)	59 (76.6%)	0 (0.0%)	77 (100.0%)
	Given 90 tablets	15 (4.7%)	307 (93.8%)	5 (1.5%)	327 (100.0%)
	Given 120 tablets	14 (4.5%)	281 (92.5%)	9 (3.0%)	304 (100.0%)
Deworming	Not given	16 (7.7%)	191 (91.3%)	2 (1.0%)	209 (100.0%)
	Given at 3rd/4th visit	40 (7.9%)	457 (89.8%)	12 (2.4%)	509 (100.0%)
Tetanus toxoid vaccine	Given one dose	12 (70.6%)	5 (29.4%)	0 (0.0%)	17 (100.0%)
	Given two doses	44 (6.3%)	643 (91.7%)	14 (2.0%)	701 (100.0%)

The mean (± SD) birth weight of neonates was 3113.0 (±474.9) gram with the minimum and maximum birth weights of 1800 and 4400 g respectively. The mean birth weight of neonates among women who received acceptable and unacceptable quality of ANC service was 3232 (± 0.478) and 3062 (± 0.476) grams. Of the total pregnant women who deliver at health facilities, 56 (7.8%; 95%CI = 6.0%, 9.7%) gave a low birth weight baby, 648 (90.3%; 95%CI = 88.0%, 92.2%) gave a baby with normal birth weight (2500-4000 g) and 14 (1.9%; 95%CI = 1.0%, 3.1%) gave birth overweight babies (> 4000 g).

### Association between contents of ANC service with birth weight

In a bivariate Generalized Estimating Equation linear regression analysis, there was a positive association between birth weight of neonates with the maternal nutritional advice during ANC, Iron –folic acid supplementation, deworming with anti-heliments, doses of tetanus toxoid vaccination of the mother, Venereal Disease Research Laboratory (VDRL) test of the mother, hematocrit test of the mother at first ANC visit, hematocrit test of the mother at fourth ANC visit, advice on Insecticide Treated Bed Net (ITN) use and most of the socio-demographic factors such as residence, maternal age, educational status, occupation and parity. Therefore, all these variables were fitted to the multivariable GEE linear regression analysis.

In multivariable Generalized Estimating Equation linear regression analysis, maternal nutritional advice during ANC, Iron supplementation, tetanus toxoid vaccination, educational status and parity remained to have statistically significant association with birth weight. In addition, deworming with anti-heliments, advice on ITN use, having VDRL test, having hemoglobin test at fourth ANC visits and living in urban areas showed an increase in birth weight but the association was not statistically significant.

When the frequency of maternal nutritional advice during ANC visits was increased by one, the birth weight of a neonate was increased by 147 g (β = 0.147, 95%CI = 0.11, 0.19). When the number of iron tablet supplementation to pregnant women during ANC visits decreased from 90 or more tablets to less than 90 tablets, the birth weight of their neonates decreased by 358 g (β = − 0.358, 95%CI = − 0.476, − 0.240). When the dose of tetanus toxoid vaccine given to pregnant women during ANC visits increased from one to two doses till delivery, the birth weight of their neonates increased by 609 g (β = 0.609, 95%CI = 0.316,0.903). When the educational status of women increased by one unit, the birth weight also increased by 79 g (β = 0.079, 95%CI = 0.06, 0.10). When the parity of women increased by one the birth weight of their neonates decreased by 174 g (β = − 0.174, 95%CI = 0.24, 0.11) (Table 4).

**Table 4:** Multivariable Generalized Estimating Equation linear regression to identify determinants of birth weight among pregnant women attending ANC at public health facilities of Bahir- Dar City Administration (*n* = 718), October 2015 to August 2016

Dummy variables		Number (%)	Un-Standardized β with 95%CI	Standardized β with 95%CI	*P*-value
Maternal nutritional advice during ANC		718 (100)	0.147 (0.11,0.19)	0.138 (0.10,0.17)	0.001*
Residence	Urban	676 (94.2)	0.398 (0.249,0.547)	0.122 (−0.02,0.26)	0.09
	Rural	42 (5.8)	0^a^	0^a^	
Iron supplementation	Took <9o tabs	87 (12.1)	−0.358 (− 0.476,− 0.240)	-0.240 (− 0.34,-0.14)	0.001*
	Took ≥90 tabs	631 (87.9)	0^a^	0^a^	
TT vaccination	Took two doses	701 (97.6)	0.609(0.316,0.903)	0.286(0.03,0.54)	0.03*
	Took one dose	17 (2.4)	0^a^	0^a^	
Deworming at 3rd/4th visit	Yes	509 (70.9)	0.109 (0.035,0.183)	0.031 (−0.04,0.11)	0.41
	No	209 (29.1)	0^a^	0^a^	
Hematocrit test at 1st visit	Yes	684 (95.3)	0.115 (−0.041,0.271)	−0.002 (− 0.15, 0.15)	0.98
	No	34 (4.7)	0^a^	0^a^	
Hematocrit test 3rd visit	Yes	378 (52.6)	0.121 (0.052,0.190)	0.026 (−0.04, 0.09)	0.46
	No	340 (47.4)	0^a^	0^a^	
VDRL test	Yes	644 (89.7)	0.120 (0.008,0.231)	0.028 (−0.08, 0.14)	0.61
	No	74 (10.3)	0^a^	0^a^	
Advice about ITN use	Yes	564 (78.6)	0.069 (−0.011,0.149)	0.031 (−0.05, 0.11)	0.42
	No	154 (21.4)	0^a^	0^a^	
Educational status of the mother		718 (100%)	0.079 (0.06,0.10)	0.064 (0.05,0.08)	0.001*
Maternal age		718 (100%)	−0.067 (−0.13,-0.01)	−0.037 (− 0.10, 0.03)	0.25
Parity		718 (100%)	−0.174 (0.24,0.11)	−0.092 (− 0.16,-0.03)	0.01*

*(indicates statistical significance at *p*-value < 0.05), 0^a^ indicates a reference category for the categorical predictor variables

*NB* The data about birth weight were entered in kilograms to the data set and the betas are converted to grams by multiplying with 1000 for the ease of interpretation

## Discussion

World Health organization set a goal to reduce the number of infants with LBW by 30% by the year 2025 and similarly the Sustainable Development Goal (SDG) by 2030 also aimed to reduce neonatal mortality to at least as low as 12 per 1000 live births. Though LBW contributes to 60%to 80% of all neonatal deaths [3, 12]; In this study about 7.8% (95%CI = 6.0–9.7% pregnant women gave low birth weight baby. This finding is consistent with other study findings in Ethiopia, 9%in urban population and 11% in the general population [2], in Ghana (6.1%) [13], in Colombia (8.7%) [14], in Mexico (8%) [15] but is lower than study findings in Angola and (25.9%) [16], in India (26.8%) [17].

The possible reason might be due to the difference in the characteristics of study participants as all the participants in this study were enrolled if they started their ANC in their first trimester and were also followed till their four antenatal visits and most (94%) were from urban and peri- urban populations that might increase the tendency to get the essential components of antenatal care like maternal nutritional advice, Iron-folic acid supplementation and other services during subsequent ANC visits that might contribute positively for birth weight. The difference in the study period might also have its own effect for the difference in the findings as many interventions like the community based newborn care packages for improving maternal and newborn health are being implemented by different countries including Ethiopia. In addition, except the current study and the study in Ghana all other studies collect information about birth weight based on mother’s recall or birth registration records that might affect accuracy of their result.

This study revealed that the frequency of nutritional advice given to pregnant women during their ANC visits have statistically significant effect on increasing the birth weight of their babies. In contrary to this finding a meta-analysis done from thirty-three studies evidenced nutrition education and counselling significantly improved mean birth weight but was not significantly associated with the risk of low birth weight [18]. The difference might be due to the reason that this study considers dose –response relationship of nutritional advice unlike those studies included in the meta- analysis and the authors of the meta- analysis also indicated except one study, the overall quality of studies extracted were low. The other possible reason could be the difference since most of the studies included in the meta-analysis were conducted in developed countries (USA, UK, Australia, Argentina and Greece). The participants of studies in these countries might have a better educational status and awareness on maternal education during pregnancy and other essential services needed for a pregnant woman so that there might not be a significant difference between women who were counselled and those who were not counselled about maternal nutrition during ANC.

In low-income countries, 25% of low birth weight was attributable to maternal anemia during pregnancy [19]. In spite of this, the current study also showed more than one in ten pregnant women were supplemented with less than 90 tablets throughout their pregnancy period. Iron-folic acid supplementation had also a dose –response relationship with birth weight; this study found that the birth weight of babies among pregnant women who were not supplemented with Iron-folic acid tablets or those who were supplemented with < 90 tablets during their ANC visits was significantly decreased compared to those who were supplemented with 90 tablets or more.

This finding is also supported with a Meta-analysis done from ten randomized control trials indicating 2 % increase in birth weight for every two-fold increase in folate intake. Other studies conducted in Spain and Atlanta also revealed duration longer than 4 months of iron supplementation gave significant protective odds ratio for low birth weight and women with low folate intake (≤240 mg/d) had > 3 times greater risk for low birth weight infant than women with folate intake > 240 μg/d respectively [20–23]. The physiological mechanism of iron supplementation on birth weight is not clearly understood, however, there are two hypotheses about improvements in birth weight due to iron supplements. First, iron deficiency anemia leads to changes in nor-epinephrine, cortisol and corticotrophin resulting in oxidative stress to fetal growth which is reduced by iron supplementation. Second, iron supplementation helps to improve appetite leading to improvement in the overall nutritional status of mother [24].

In this study majority (97.6%) of pregnant women were given two doses of tetanus toxoid vaccinations compared to the Ethiopian Demographic and Health Survey report in 2016 revealed that only half of the pregnant women who gave birth 5 years prior to the survey took two doses (with 72% urban and 46% rural women) [25]. The current study also showed providing two doses of tetanus toxoid for pregnant women during their antenatal care visits significantly increased the birth weight of their babies compared to those women who were given only one dose. The increase in birth weight of babies born from women who received two doses of tetanus toxoid might not be due to the direct effect of tetanus toxoid vaccination on birth weight rather it might be due to the fact that when women came to health facility for TT vaccinations, they are more likely to get nutritional advices or iron- folic acid supplementations that contribute for better birth weight.

This study also found that there was an increase in birth weight as educational status of pregnant women increased from no education or primary education to secondary education and above. This is also supported by studies conducted in Bangladesh and Beirut [26, 27]. In contrary to maternal education that had positive association to birth weight, multi parity or grand multi parity of mothers and older age of the mother (≥ 35 years) had negative association with birth weight.

Prospective follow up of pregnant women through observation while they get the ANC service; collecting information about birth weight of the neonate at the moment of birth and applying a generalized estimating equation analysis to control the cluster effect of women who received the ANC service in the same health facility could be taken as the strength of the study but excluding women who gave birth at home due to missing of birth weight data and Hawthorn effect could be the limitation as those women might have different characteristics.

## Conclusion and recommendations

The study found that near to one in ten neonates were born with low birth weight. Access to quality antenatal care increases the birth weight of new born babies. Essential components of antenatal care (maternal nutritional advice, iron-folic acid supplementation, and tetanus toxoid vaccination) and socio- demographic variables like maternal educational status and parity were determinants for birth weight. Strengthening adherence of providers to essential components of antenatal care like nutritional advice, iron- folic acid supplementation and tetanus toxoid vaccination for every pregnant woman through regular monitoring and provision of feedback and need based capacity building is very important for reducing the risk of low birth weight.

## Abbreviations

ANC: Antenatal Care
CI: Confidence Interval
FANC: Focused Antenatal Care
GEE: Generalized Estimating Equation
ITN: Insecticide Treated Bed Net
LBW: Low Birth weight
SPSS: Statistical Packages for Social Sciences
TT: Tetanus Toxoid
VDRL: Venereal Disease Research Laboratory
